# Acetabular defects in revision hip arthroplasty: a therapy-oriented classification

**DOI:** 10.1007/s00402-020-03379-6

**Published:** 2020-02-25

**Authors:** Dieter Christian Wirtz, Max Jaenisch, Thiemo Antonius Osterhaus, Martin Gathen, Matthias Wimmer, Thomas Martin Randau, Frank Alexander Schildberg, Philip Peter Rössler

**Affiliations:** Department of Orthopaedics and Traumatology, University Hospital Bonn, Germany, Sigmund-Freud-Straße 25, 53127 Bonn, Germany

**Keywords:** Hip, Acetabulum, Revision, Arthroplasty, Classification, Paprosky

## Abstract

**Introduction:**

The treatment of severe acetabular bone loss remains a difficult challenge. No classification system is available that combines intuitive use, structured design and offers a therapeutic recommendation according to the current literature and modern state of the art treatment options. The goal of this study is to introduce an intuitive, reproducible and reliable guideline for the evaluation and treatment of acetabular defects.

**Methods:**

The proposed Acetabular Defect Classification (ADC) is based on the integrity of the acetabular rim and supporting structures. It consists of 4 main types of defects ascending in severity and subdivisions narrowing down-defect location. Type 1 presents an intact acetabular rim, type 2 includes a noncontained defect of the acetabular rim ≤ 10 mm, in type 3 the rim defect exceeds 10 mm and type 4 includes different kinds of pelvic discontinuity. A collective of 207 preoperative radiographs were graded according to ADC and correlated with intraoperative findings. Additionally, a randomized sample of 80 patients was graded according to ADC by 5 observers to account for inter- and intra-rater reliability.

**Results:**

We evaluated the agreement of preoperative, radiographic grading and intraoperative findings presenting with a *k* value of 0.74. Interobserver agreement presented with a *k* value of 0.62 and intraobserver at a *k* value of 0.78.

**Conclusion:**

The ADC offers an intuitive, reliable and reproducible classification system. It guides the surgeon pre- and intraoperatively through a complex field of practice.

## Introduction

The operative treatment of severe acetabular bone loss remains one of the greatest challenges facing the field of revision hip arthroplasty. Considering the rising number of primary total hip arthroplasties, which have reached 3.2 million per year in Europe alone, there is an increased probability of component loosening associated with periprosthetic bone defects [1]. With the age of the general population increasing revision rates of up to 12% will be common [2].

General principles in the treatment of severe periacetabular bone loss include the reconstruction of a physiological joint geometry, including correct positioning of the anatomical hip center of rotation, as well as an appropriate femoral offset [3]. A primary stable implantation with proper force transmission to the remaining acetabular bone stock is essential to enable long-term stability. Noncontained acetabular defects should be transitioned to contained defects using some form of augmentation. Another goal of hip revision arthroplasty is the downsizing of the bone defect to improve the outcome in following revision procedures [4].

To achieve these goals, precise preoperative planning and proper implant choice are essential. Because correct interpretation of radiographic findings can be difficult an exact definition of common patterns of defect morphology with a resultant therapeutic recommendation is needed.

To address this purpose, a number of classification systems have been published, which can in general be divided into 2 categories. Classifications such as proposed by the AAOS or Engh and Gross follow a volumetric approach and offer a rather simplified, but reproducible evaluation, when in contrast classifications, such as proposed by Paprosky, Saleh or D’Antonio focus on detailed defect description and preoperative planning [5–9]. Due to the simple nature of the first category, these classifications lack therapeutic guidance while the second category can be difficult to memorize and therefore impede intuitive use.

To the authors knowledge, no classification system is available that combines intuitive use and structured design while still producing a detailed defect description and offering a therapeutic method according to the current literature and modern state of the art treatment options.

The goal of the present retrospective study is to introduce an intuitive guideline for the evaluation and treatment of acetabular defects in revision hip arthroplasty. To account for reliability, we compared preoperative gradings with intraoperative findings and in the evaluation of reproducibility we rated inter- and intra-rater agreement.

We hypothesized that the ADC is a reliable and reproducible classification system, which provides the surgeon preoperativley with a valid estimation of the defect severity and the needed preparations. Additionally, we devised a clear algorithm for intraoperative assistance while deciding the definitive choice of implants and additions such as augments or bone grafting.

## Materials and methods

### Study design and patients

Study approval was obtained through our institution’s review board. The presented single-centre, cohort study was based on retrospectively collected data of 253 consecutive patients, which underwent acetabular revision surgery between 2011 and 2017 for any reason. Inclusion criteria were cases of THA revision for any reason with exchange of at least the acetabular component. Patients were excluded if the preoperative radiographs could not be retrieved or were of insufficient quality, as well as if intraoperative recordings were incomplete and did not allow for a reliable grading. In addition, patient characteristics including age, sex, date of revision and implant inserted were collected. During the selection process, 21 cases were excluded because no digital copies of preoperative radiographs could be retrieved or because radiographs were of insufficient quality. Additionally, 25 cases were excluded because intraoperative data did not match the defined requirements (see intraoperative evaluation). After exclusion 207 patients have been evaluated.

### Intraoperative evaluation

Collection and conversion into ADC grading of intraoperative data was carried out in a retrospective manner by one of the originators of the ADC. Surgical reports were screened for information concerning the exhibited acetabular defect location and size. Additional information, such as implants used and/or use of augmentation were taken into consideration as well. Cases were excluded if the exact defect location was not specified and if no measurement of defect size had been recorded.

### Radiographic evaluation

The radiographs were anonymized by numerical coding and all identifying features have been removed. Subsequently, the radiographs were graded according to ADC by one of the originators of the ADC, without prior knowledge of the recorded intraoperative findings. As a next step, agreement of radiographic and intraoperative grading using Cohens $\kappa$ has been performed.

After power analysis, 80 anonymized radiographs were chosen at random and distributed to 5 raters. All of these raters were experienced orthopaedic surgeons in the field of hip revision arthroplasty. Each rater received 1 teaching session consisting of thorough explanation of the classification system and supervised evaluation of 10 random cases. The teaching session did not include any of the 80 cases used for later evaluation. A scoring sheet inspired by a publication of Yu et al. and adapted for the ADC has been distributed to observers [5]. None of the raters had any prior knowledge of the ADC or were involved in the creation. The distributed radiographs have been analysed on 2 occasions with an interval of at least 2 weeks in between as a washout period. Radiographs were relabelled and randomized prior to the second evaluation.

The preoperative radiographs included pelvis a.p. standing and involved hip axial. The preoperative images were graded according to ADC using IMPAX EE (Agfa HealthCare GmbH, Bonn, Germany).

### Classification system

The ADC is based on the integrity of the acetabular rim and the supporting structures. It consists of 4 main types of defects ascending in severity, with an additional subdivision into a, b and c narrowing down-defect location.

### Type 1 defects

**Fig. 1:**
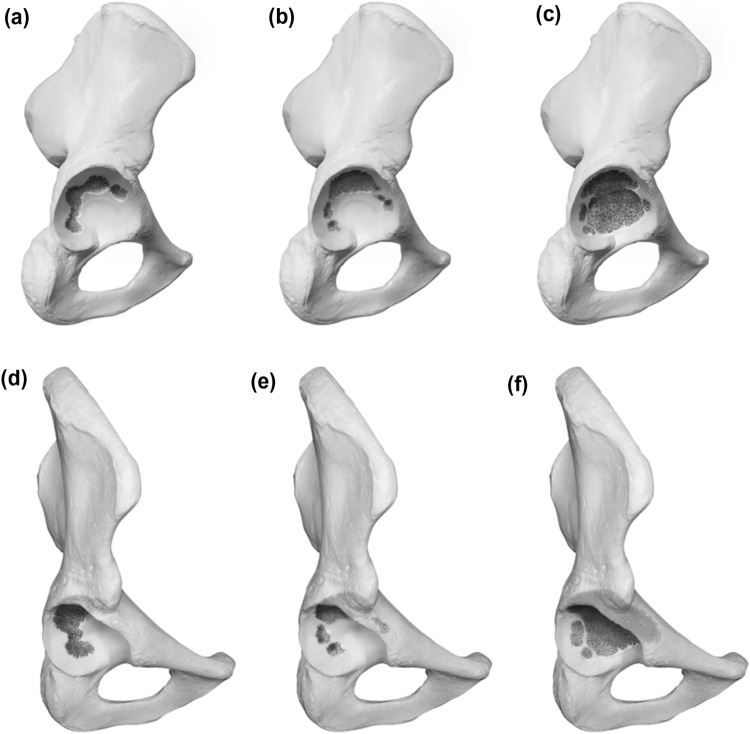
** a**–**f** Acetabular Defect Classification (ADC) Type 1 **a** lateral view of a Type 1a defect **b** lateral view of a Type 1b defect **c** lateral view of a Type 1c defect **d** 45° view of a Type 1a defect **e** 45° view of a Type 1b defect **f** 45° view of a Type 1c defect

Type 1 defects are characterized as contained defects with the acetabular rim remaining intact and the acetabulum only showing cancellous bone defects in different locations according to subdivision. A 1A defect displays randomly distributed cancellous defects, which respect the superomedial aspect of the acetabulum and the medial wall. A 1B defect exhibits a lysis of the superomedial aspect of the acetabulum in addition to defects already described for A. A 1C defect displays a deficiency of the medial wall, which does not affect the anterior or posterior column. Graphic illustrations are presented in Fig. 1.

### Type 2 defects

**Fig. 2:**
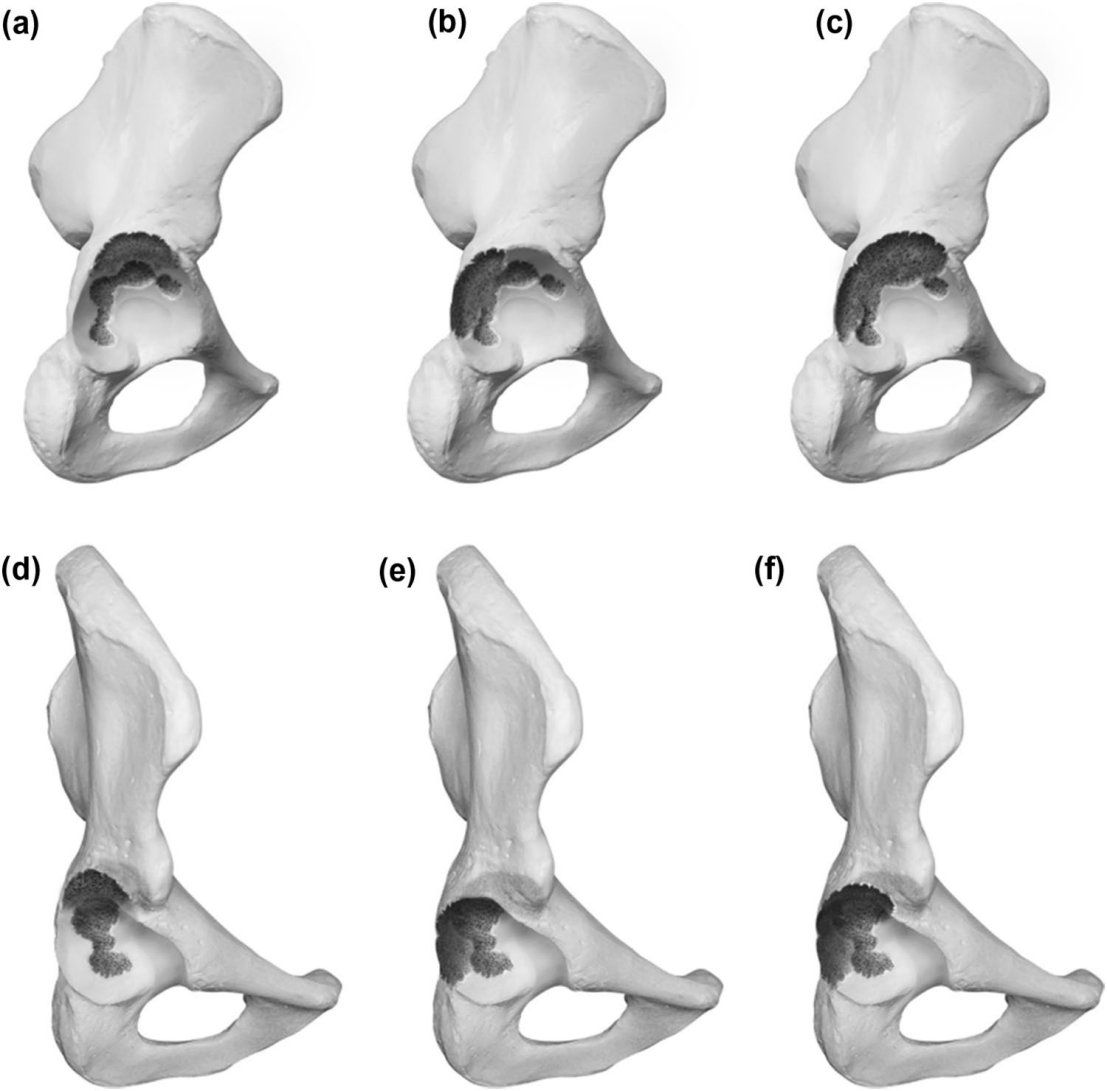
**a**–**f** Acetabular Defect Classification (ADC) Type 2 **a** lateral view of a Type 2a defect **b** lateral view of a Type 2b defect **c** lateral view of a Type 2c defect **d** 45° view of a Type 2a defect **e** 45° view of a Type 2b defect **f** 45° view of a Type 2c defect

Type 2 demonstrates a noncontained defect of the acetabular rim in addition to cancellous bone defects. The defect measures below or equal 10 mm in the vertical plane and is considered as nonstructural. For 2A the rim defect affects the superolateral portion while in 2B the posterior column is deficient (horizontal plane). A Type 2C defect is a combination of A and B and displays a defect including the full weight bearing portion of the rim. Because of its measurement below 10 mm, it is also considered as nonstructural. Graphic illustrations are presented in Fig. 2.

### Type 3 defects

**Fig. 3:**
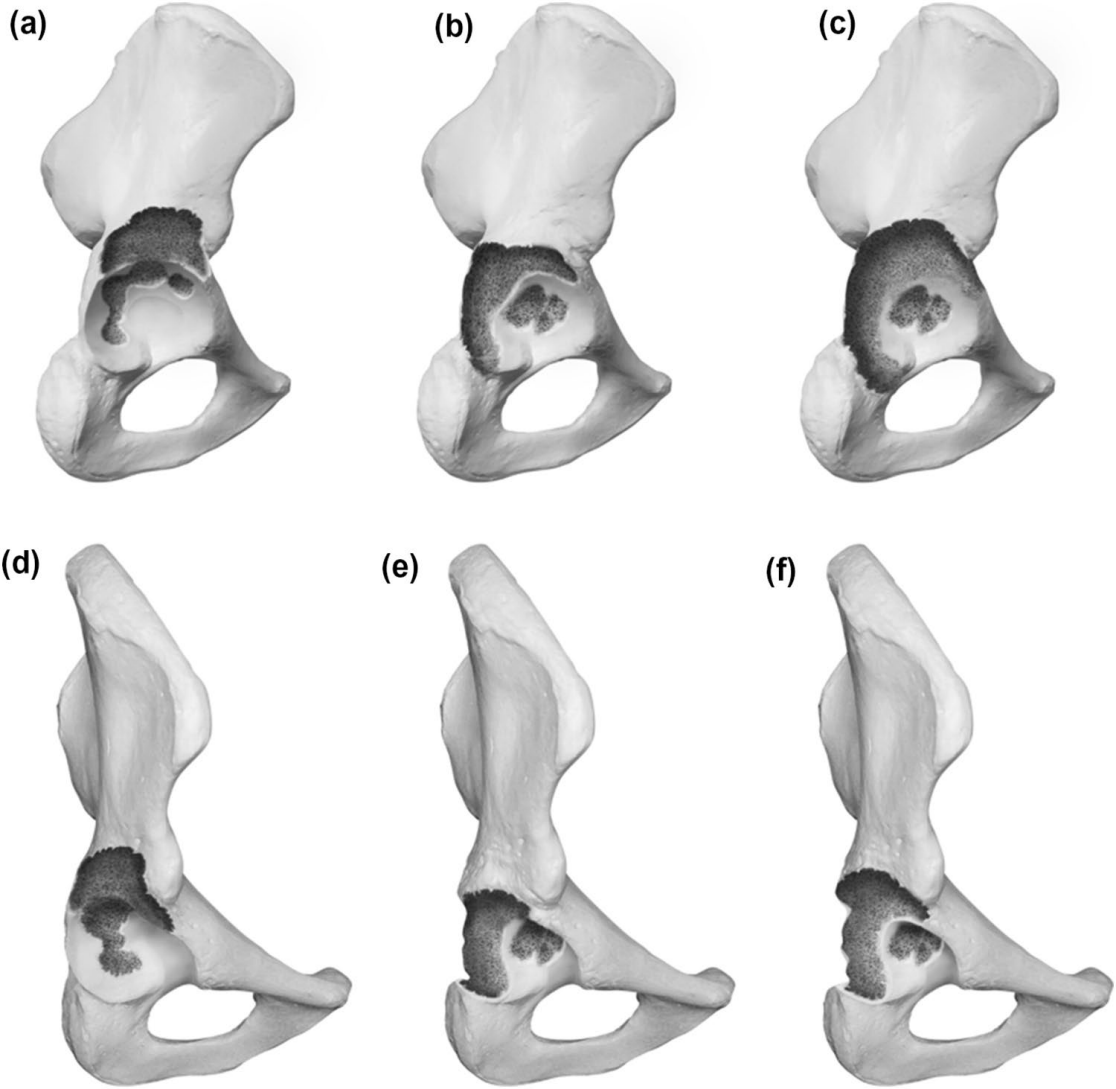
** a**–**f** Acetabular Defect Classification (ADC) Type 3 **a** lateral view of a Type 3a defect **b** lateral view of a Type 3b defect **c** lateral view of a Type 3c defect **d** 45° view of a Type 3a defect **e** 45° view of a Type 3b defect **f** 45° view of a Type 3c defect

Type 3 defects possess noncontained, structural defects of the acetabular rim over. The subdivision follows the same structure as for the type 2 defects with A including the superior aspect, B the posterior column and C being a combination of both. Graphic illustrations are presented in Fig. 3.

### Type 4 defects

**Fig. 4:**
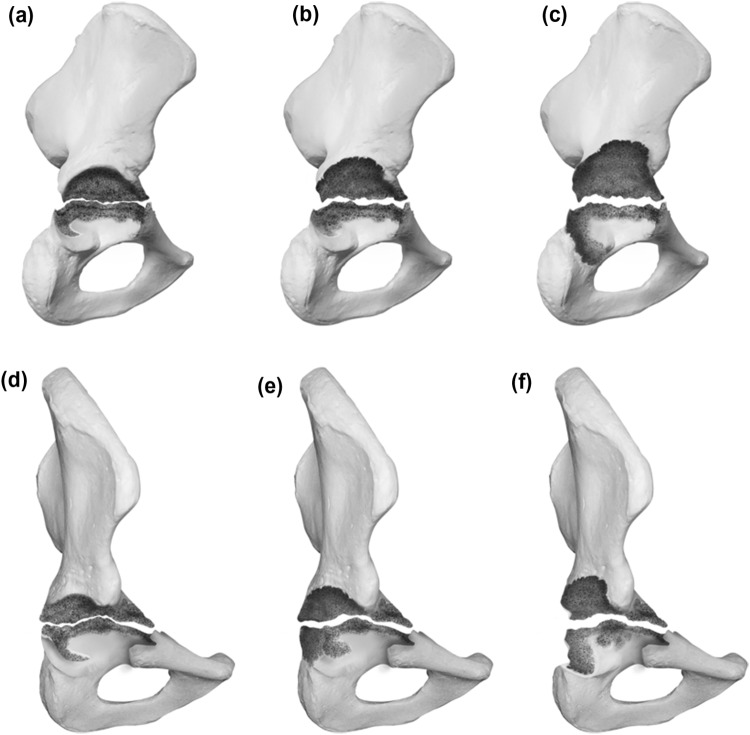
**a**–**f** Acetabular Defect Classification (ADC) Type 4 **a** lateral view of a Type 4a defect **b** lateral view of a Type 4b defect **c** lateral view of a Type 4c defect **d** 45° view of a Type 4a defect **e** 45° view of a Type 4b defect **f** 45° view of a Type 4c defect

Type 4 defects exhibit a disruption of the bone stock between the ischium and the ilium. The anterior and posterior columns are rendered nonsupportive. The subclassification in A, B and C account for the amount of remaining superior bone stock. For A the superior bone stock is considered supportive, for B a nonstructural superior rim defect under/equal 10 mm in the vertical plane is described and for C a structural superior rim defect over 10 mm accompanies the pelvis discontinuity. Graphic illustrations are presented in Fig. 4.

### Statistical analysis

All analyses were performed using IBM SPSS Statistics 1.0.0.1131 (IBM Inc., Armonk, New York, USA). The level of significance was set at *p* < 0.05. The confidence interval has been set at 95%. Fleiss $\kappa$was used as a means to account for inter-rater reliability in the process of comparing ordered categorical data with more than 2 observers. Intra-rater reliability was being accessed using Cohens $\kappa$and in a next step producing the mean $\kappa$of all raters. The extend of agreement was interpreted using the criteria described by Landis and Koch [6]. Therefore, if the $\kappa$value exceeds 0.80 excellent agreement is achieved, between 0.61 and 0.8 indicated good agreement, a score of 0.41–0.60 indicated moderate agreement, a score between 0.21 and 0.4 indicates fair agreement and finally a score of 0.20 and below indicates poor agreement.

## Results

**Fig. 5:**
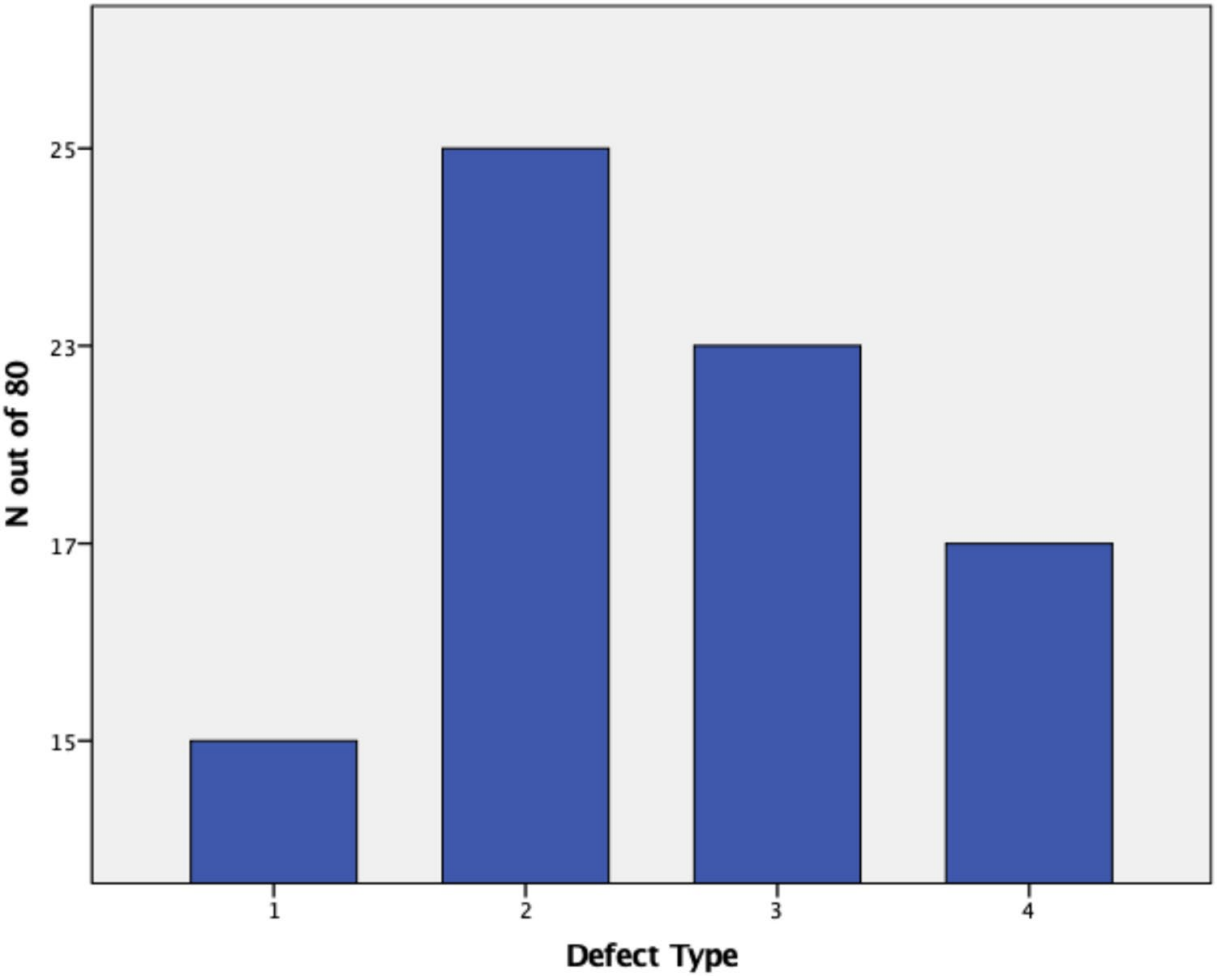
Illustration of the distribution of types of defect in the randomized sample (*y* axis *n* out of 80, *x* axis type of defect form 1–4)

In this study, 207 preoperative radiographic gradings have been compared to intraoperative findings to account for agreement of defect severity. A Cohens $\kappa$of 0.74 could be evaluated, accounting for good agreement between preoperative radiographs and intraoperative findings. The large sample of 207 proved well balanced, displaying 81 type 1 defects (39%), 72 type 2 defects (35%), 37 type 3 defects (18%) and 17 type 4 defects (8%). Due to randomization, the smaller sample to account for inter- and intra-rater reliability displayed a different distribution (Fig. 5).

**Fig. 6:**
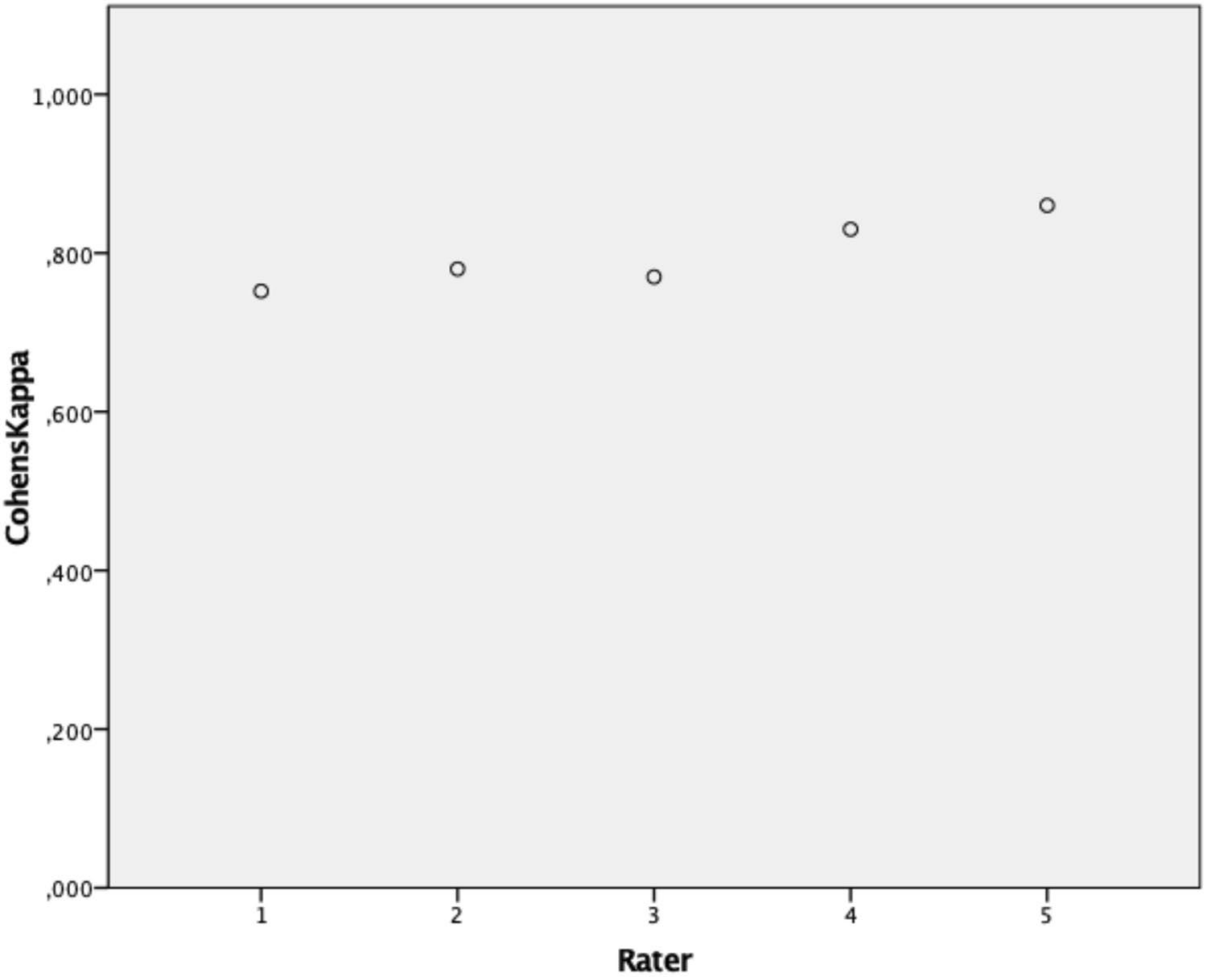
Accounting for Intrarater reliability Cohens $\kappa$ (*y* axis) has been evaluated and illustrated matching each rater (*x* axis)

To account for inter-rater reliability, 80 patients have been evaluated by 5 different observers. Testing for inter-rater reliability, a Fleiss $\kappa$of 0.624 (low CI 0.6; high CI 0.65) could be evaluated falling into the good agreement range as defined by Landis and Koch [6]. When testing for intra-rater reliability, Cohens $\kappa$of each of the 5 raters has been analyzed and the mean was calculated (Fig. 6). Testing for intra-rater reliability revealed a mean $\kappa$of 0.79, which still remains to be in the good agreement range almost accounting for perfect agreement.

## Discussion

Acetabular revision arthroplasty provoked by extensive periprosthetic osteolysis presents a difficult challenge for orthopaedic surgeons. Depending on the defect size and location different approaches and implants, as well as additions like augments or bone grafting, need to be considered. In such a high stakes field of surgery, meticulous preoperative planning is essential for achieving successful and lasting fixation.

By creating the ADC, we aim to distribute a reliable, reproducible and intuitive classification system to aid surgeons in navigating a difficult field of practice by improving the diagnostic assessment and providing therapeutic guidance.

In the creation of an ideal acetabular defect classification certain key points should be considered. First, it should be applicable to the evaluation of native radiographs of the pelvis. Patients with significant osteolytic lesions can remain asymptomatic as long as the fixation is stable. During those asymptomatic years, bone loss progresses and increases the difficulty of revision options. To achieve an early diagnosis of increasing acetabular osteolysis, regular, postoperative follow-up assessments, including plain radiographs of the pelvis are essential. Native radiographs, present a limited amount of radiation exposure, are cheap, easy to produce and widely available.

The radiographic evaluation according to the ADC appears to provide a reliable estimation of the defect extend compared to intraoperative findings with a *k* value of 0.74 indicating good agreement. The authors contribute the good results to the analytical approach of the provided evaluation spreadsheet, which focuses on standardized definitions of radiographic landmarks and a structured analysis. Each supporting structure is being evaluated separately and added up to provide a final grading.

The analysis of the discrepancies between preoperative grading and intraoperative situation showed a limited accuracy regarding defects of the posterior column and wall, as well as the recognition of pelvic discontinuities. This problem has been described before and in most cases, it is due to radiopaque implants obscuring the visualization in plain radiographs [5]. A publication by Claus et al. evaluated 128 radiographs, and was able to report a decent sensitivity for defects of the ilium (71.5%), but a poor sensitivity for lesions of the posterior column (15%) [7]. Because the posterior column/wall is essential for proper implant choice, a computer tomography should be performed preoperatively if the integrity of the dorsal structures is in question [8]. Some studies also show an increased sensitivity for the recognition of posterior wall/column defects and pelvic discontinuity through the use of oblique radiographs [9, 10].

Secondly in order for a classification to integrate itself in the daily use of a clinician, it needs to be intuitive. While creating the ADC, the authors decided to use a detailed defect description in order to offer a clear therapeutic algorithm, but at the same time kept definitions and structure consistent throughout the system to facilitate intuitive application. The 4 main categories are easy to memorize and already provide vital information of the defect morphology. Dividing defects due to integrity/extend of damage to/of the acetabular rim and presence of pelvic discontinuity already sets the tone for possible therapeutic options. The subcategories further narrow down-defect location and complete the necessary evaluation to apply the therapeutic algorithm.

The practical application of a classification only finds its way in daily practice and research if a sufficient reproducibility and reliability can be established.

Traditional classification systems, such as AAOS, Paprosky et al. or Gross et al. have yielded disappointing results in evaluations in literature and appear to mostly fall into the poor to moderate range for intra-rater and inter-rater reliability when evaluating preoperative radiographs [6, 8, 11]. As discussed above, correct interpretation of radiographic signs can be challenging and presents a limited reliability in itself [5, 7]. In a study conducted by Yu et al. a significant improvement of the agreement concerning the Paprosky classification could be observed when utilizing teaching sessions and a structured scoring table guiding evaluation [5]. The inter-rater and intra-rater reliability of the ADC presents with *k* values of 0,62 and 0,78 as satisfactory falling into the good agreement category as defined by Landis and Koch [6]. The authors contribute this to the overall structured and intuitive design, as well as to the utilization of teaching session and a structured scoring table. Since this publication represents the introduction of the ADC, more research into the reliability and reproducibility is needed, but these early results are promising and confirm this new classification as reliable.

The final and most important criteria for a well-established classification is a clear therapeutic algorithm, which moves along the different gradings. Established classification systems, such as provided by Paproksy et al. focus on the utilization of bulk allografts, which have been established as having a poor long-term stability as discussed below [12]. Since most established defect classifications have been introduced in the 1990s, an update, which includes and addresses the application of modern techniques is needed.

The following treatment choices are recommendations based on the algorithm the authors established and use in their own clinical practice and a thorough review of the current literature. The full algorithm is provided in Table 1.

**Table 1:** Therapeutic recommendation based on defect type according to ADC

Type of defect	Implant choice
1 A/B	Pressfit cup/Screw-in cup Impaction bone grafting of the medial and superomedial aspect of the acetabulum
1 C	Pressfit cup/Cup and Cage/Modular cage/Screw-in-cup
	Impaction bone grafting of the medial and superomedial aspect of the acetabulum
2 A/B/C	Metal-Augmentation of defect through: A: Augment-and-Cup/Augment-and-(modular)-Cage/Oblong Cup/Cranial socket system; B/C: additional flanges and/or iliac peg Impaction bone grafting of the medial and superomedial aspect of the acetabulum
3 A/B/C	Metal-Augmentation of defect with additional flanges through: Augment-and-(modular)-Cage Impaction bone grafting of the medial and superomedial aspect of the acetabulum
4 A	Iliac–ischial plating in combination with: Augment-and-(modular)-cage – Oblong cup/Cranial socket system with iliac peg and additional flanges Impaction bone grafting of the medial and superomedial aspect of the acetabulum
4 B	Augment-and-(modular)-cage, oblong cup with iliac peg and additional flanges, Custom individualized monoblock pelvic replacement with tripolar cup system (dual mobility) Impaction bone grafting of the medial and superomedial aspect of the acetabulum
4 C	Custom individualized monoblock pelvic replacement with tripolar cup system (dual mobility) Impaction bone grafting of the medial and superomedial aspect of the acetabulum

Type 1 defects are defined by an intact acetabular rim with cancellous bone defects in different locations. Therefore, a circumferential pressfit can be established [13]. Additionally, screws can be added to the implant of choice, but only if a primary stable fixation is achieved through pressfit. In the interest of defect downsizing, impaction bone grafting should be applied. Several publications report good mid- and long-term results and the observation of bone mineral density changes, which can be interpreted as a progressive apposition of vital new host bone [4, 14, 15]. Impaction bone grafting has proven itself to be a valid method of treatment and should be used in contained defects or severe defects in combination with other methods of reconstruction. If the lesion of the medial wall in 1C defects is rather large causing a protrusion medially, a cup-and-cage-design can be used to bridge the defect and allow for proper grafting and defect downsizing [16, 17]. However, there are publications showing good short-term results when treating protrusion acetabuli relying on rim pressfit even in large medial defect situations [18]. Therefore a screw-in-cup is feasible as well. 

Once the defect level reaches 2 or above a noncontained defect of the acetabular rim exists. In the authors opinion, a long-term stable fixations and anatomical reconstruction of the center of rotation can only be achieved by converting the uncontained defect into a contained defect through augmentation. Different means of augmentation have been introduced and are discussed controversially. The superolateral portion of the acetabulum takes up a special role for being the most taxed portion of the rim considering weight bearing and therefore often exhibits a pronounced sclerosis and limited vascularization. Therefore, structural bulk allografts do not present a promising option with limited integration, consecutive resorption and failure of fixation in the long term [19]. Various publications were able to display a high rate of failure in long-term follow-up in strong contrast to promising short-term results [20, 21]. Even in radiographically stable allografts, a fibrous encapsulation without significant bone union and no evidence of implant ingrowth could be evaluated postmortem [22].

Means of augmentation can include oblong cups, cranial socket systems, augment-and-cup and augment-and-cage designs. Recently, promising early results for an augment-and-modular-cage-design have been published showing an improvement of clinical function and patient reported outcome as well as efficient reconstruction of the acetabular center of rotation [23]. In cases of severe bone loss to the dorsal column anatomic flanges and/or iliac pegs should be added to the construct.

Once the defect size exceeds 10 mm and is therefore defined as a type III defect additionally to the augmentation an anatomic flange is necessary even for isolated superolateral defects (A) and should be added to the construct in order to bridge the defect and obtain contact to a sufficient amount of stable host bone for proper fixation. This can be applied through a conventional or modular augment and cage system. Oblong cups or cranial socket systems are no longer feasible because the extensive craniolateral defect leads to consecutive cranialization of the hip center of rotation if not adequatly augmented.

Pelvic discontinuity presents the most severe defect situation portrayed in this classification. It poses a most difficult challenge to the surgent and implant and needs to be evaluated and planned carefully to allow for successful reconstruction. Independently of the chosen method, the goal is to achieve a stable fixation and anatomical reconstruction of the center of rotation as well as the healing of the discontinuity [24].

Depending on the remaining bone stock, different operative approaches can be chosen. Even after careful preparation, enough bone stock remains to allow for a stable bridging and an extramedullary iliac–ischial plate can be applied effectively downsizing the type IVa defect, which then can be treated accordingly taking the remaining superior and dorsal bone stock into consideration [25, 26].

If plate fixation does not pose a feasible possibility for defect downsizing, a primary stable fixation needs to be achieved through implant design. Different implant designs offer a combination of an intramedullary iliac peg, an anatomic flange and different means of defect augmentation [27, 28]. Those systems are applicable up to IV B defects, while in IV C defects due to the massive amount of bone loss, stable fixation as well as anatomical reconstruction of the center of rotation are unlikely.

If the pelvic osteolysis reaches a type IV C defect, the authors recommend the use of a custom-made acetabular component based on a CT scan. The continuous improvement in the recognition of pelvic osteolysis through advanced CT hardware and progress in the development of promising software postprocessing implements a precise preoperative planning and visualization of the defect morphology [8]. This enables the surgeon to utilize the remaining landmarks and apply different fixation techniques, such as flanges, intramedullary pole screws and integrated augments to properly address the specific defect configuration. Even though the amount of literature reporting on long-term outcome is still limited, and promising short-term results have been published [29–35]. Whenever the risk of gluteal insufficiency becomes apparent tripolar cup systems (dual mobility) should be utilized.

The main limitation of this study is the retrospective assessment of intraoperative defects. Due to this method, information is bound to be lost and validation is limited. In order to provide a more valid estimation of the reliability of the ADC, further studies including prospective intraoperative assessment in comparison to preoperative imaging are needed. Another factor accounting for a possible lack of reliability between preoperative grading and intraoperative findings is a limited reliability of radiographic landmarks and signs as discussed above. Along the foreseen future advancements of three-dimensional diagnostic solutions, the ADC can be applied as well.

A limitation afflicting all classification systems with a therapeutic recommendation is the anticipated further advancement of treatment options and operative techniques. For this reason, sooner or later, an updated therapeutic spread sheet will be necessary. The classification in itself, being based on biomechanical principles, will remain solid even once therapeutic possibilities evolve.

This publication focusses on the reliability and the reproducibility of the ADC. Even though a therapeutic algorithm has been provided, it is based on the current literature and expert opinion. Differences in the outcome were not being explored, and further prospective randomized studies are needed to establish a benefit in improved outcome.

## Conclusion

With the Acetabular Defect Classification (ADC), the authors introduced an innovative, reliable and reproducible classification system, which allows for detailed preoperative planning. It can be applied as a preoperative planning tool, as well as a means for step-by-step intraoperative guidance. In addition, a therapeutic guidance according to the current literature and expert opinion has been supplied.
